# Large language models in adolescent suicide prevention: from language signals to accountable action

**DOI:** 10.3389/fpubh.2026.1872450

**Published:** 2026-08-03

**Authors:** Man Li, Wendi Huang, Jingjing Wu, Nanyu Kuang, Yong Luo, Juan Huang, Nanqu Huang

**Affiliations:** 1 Department of Psychiatry, Jiaxing Kangci Hospital, Jiaxing, Zhejiang, China; 2 Department of Neurology, Third Affiliated Hospital of Zunyi Medical University (The First People's Hospital of Zunyi), Zunyi, Guizhou, China; 3 Institute of Science and Technology for Brain-Inspired Intelligence, Fudan University, Shanghai, China; 4 Neuroimaging Research Branch, National Institute on Drug Abuse, National Institutes of Health, Baltimore, MD, United States; 5 Key Laboratory of Basic Pharmacology and Joint International Research Laboratory of Ethnomedicine of Ministry of Education, Zunyi Medical University, Zunyi, Guizhou, China; 6 National Drug Clinical Trial Institution, Third Affiliated Hospital of Zunyi Medical University (The First People's Hospital of Zunyi), Zunyi, Guizhou, China

**Keywords:** adolescent suicide prevention, artificial intelligence ethics, clinical decision support, large language models, natural language processing

## Abstract

Adolescent suicide risk is often expressed through language before it becomes visible in clinical encounters, yet current prevention still relies heavily on episodic screening and limited specialist resources. Large language models (LLMs) may help interpret crisis chats, clinical notes, school-counseling records, and online disclosures, but their relevance to public mental health depends on a practical question: can language signals lead to timely and accountable human action? This mini review examines LLMs in adolescent suicide prevention through this Evidence-to-Action perspective. Current evidence is strongest for supervised risk-signal flagging and more preliminary for context summarization and extraction of suicide-related information from unstructured text. Evidence is much weaker for autonomous generative crisis intervention, especially for adolescents. The main barriers are not only technical accuracy, but also adolescent-specific validation, privacy and consent, cultural variation in distress expression, unsafe generated responses, and unclear responsibility after an algorithmic alert. We argue that near-term deployment should remain under human review and should be judged by whether it improves review timeliness, proportional escalation, safety planning, linkage to care, and equity. LLMs may contribute to adolescent suicide prevention only when embedded in clinically accountable and ethically governed systems. As a focused mini review, this paper maps conceptual boundaries and evidence gaps rather than proposing a validated deployment protocol.

## Introduction

Suicide prevention in adolescence remains difficult because risk often changes faster than clinical systems can detect. Suicidal thoughts and behaviors may intensify or recede over short periods, and adolescents do not always disclose distress during routine encounters (1, 2). Their risk is shaped by multiple social, developmental, and environmental factors (3–5).

Prediction has also remained weaker than expected. Traditional risk factors have limited prospective accuracy, and many suicide prediction models are constrained by heterogeneous outcomes, rare event rates, and insufficient external validation (6, 7). Machine learning models using electronic health records (EHRs), claims data, or longitudinal clinical variables have improved risk stratification in some settings (8–10). Yet structured data often miss the language through which adolescents express shame, hopelessness, ambivalence, perceived burdensomeness, or indirect farewell messages. Ecological momentary assessment and real-time monitoring can partly address this gap, but they also increase demands on consent, privacy protection, and response capacity (11, 12).

Large language models (LLMs) have therefore attracted attention in suicide prevention because many risk signals first appear as words. Crisis chats and other data may contain missing information from structured fields. However, the term “LLM” is often used too broadly. We distinguish encoder-based transformer models (e.g., BERT-like classifiers), which detect or classify suicide-related language, from generative large language models (e.g., GPT-type systems), which produce text. These two classes differ in evidence base, safety risk, and clinical responsibility. Generative LLMs may summarize context, support clinician- or counselor-facing drafts, explain risk factors, or assist protocol-based resource navigation under human review. Retrieval-augmented systems may ground outputs in approved protocols. Evidence from natural language processing or encoder-based classifiers supports the feasibility of language-based risk detection (13), yet this should not be treated as direct evidence that generative LLMs are ready to manage adolescent crises (14, 15).

Recent reviews have mapped artificial intelligence and LLM applications in suicide prevention, including risk detection, chatbot responses, clinical documentation, and online monitoring (13–15). A structured comparison of these reviews with the present work is provided in Supplementary Table 1. These syntheses cataloged applications across general populations without grading evidence strength for adolescent language tasks or linking model outputs to implementation safeguards. Their contribution is important, but two gaps remain especially relevant for adolescent public mental health. First, adolescents are often treated as a subgroup of general suicide prevention, although their developmental autonomy, privacy expectations, family involvement, and patterns of distress expression differ from those of adults. Second, model performance is often discussed separately from the actions that follow an alert.

**Figure 1:**
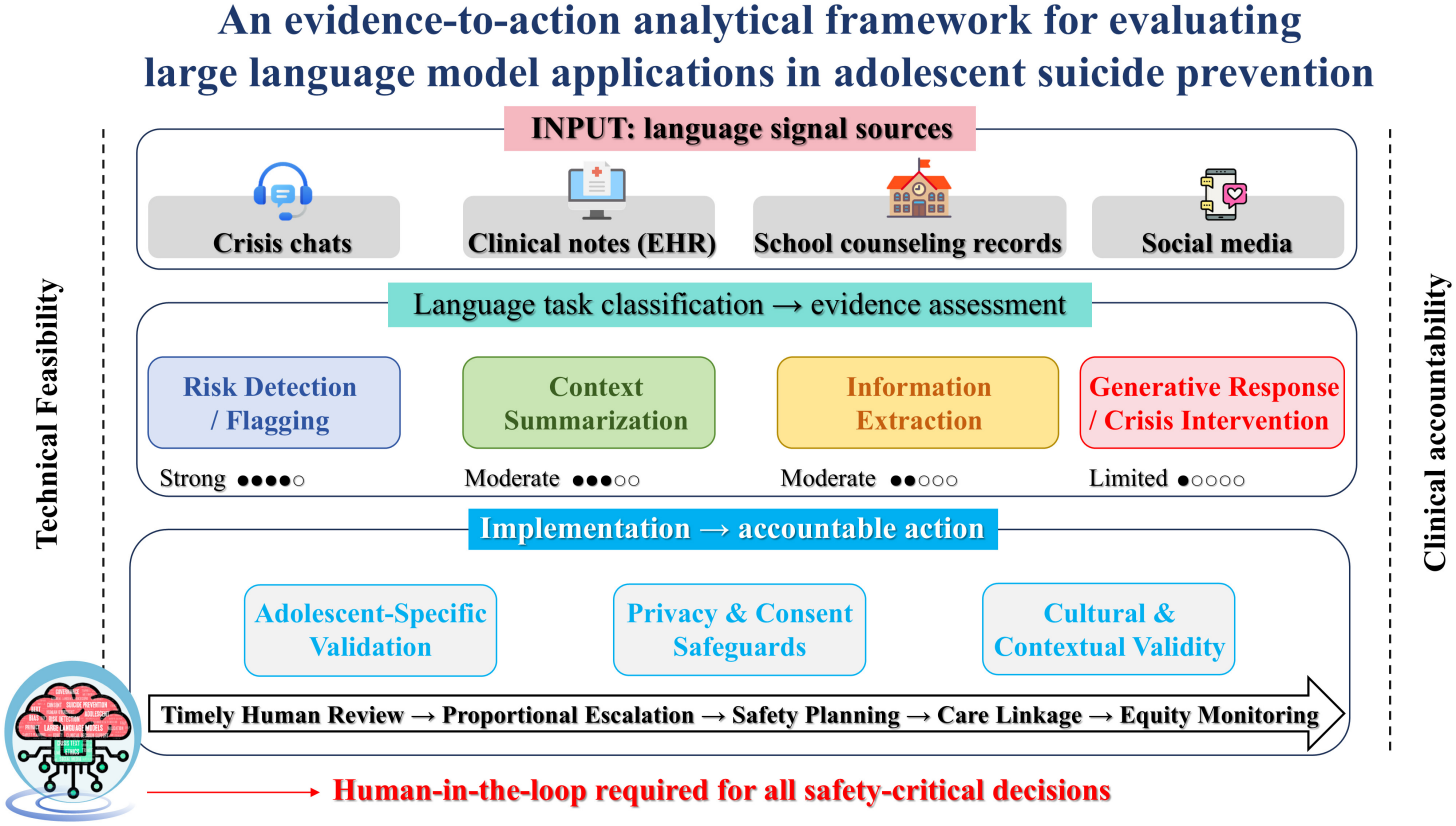
Overview of LLM-supported adolescent suicide prevention across crisis, clinical, school, online, and family contexts. Crisis (16, 17); Clinical (19–22); School (23–25); Online (29–32); Family (47–49). Created with Microsoft PowerPoint and Weiciyun. Icons by Freepik from Flaticon, licensed under Flaticon License.

This mini review therefore focuses on the path from language signals to accountable action. We examine current evidence for language-based detection and support, implementation barriers that determine whether model outputs can be translated into safe human response, and governance conditions needed for privacy, consent, equity, escalation, and responsibility. This approach moves the discussion toward the more important question of whether LLMs can improve timely, fair, and clinically accountable prevention (Figure 1). In this review, “adolescents” primarily refers to individuals aged 10–19 years; when evidence comes from broader youth or young-adult samples, it is treated as adjacent rather than directly adolescent-specific evidence.

## Review approach and analytical framework

This targeted narrative mini review was developed through purposive literature selection across PubMed, PsycINFO, Web of Science, IEEE Xplore, and arXiv from January 2023 to June 2026. Search terms combined suicide-related, adolescent, and language-model concepts. We included empirical studies and reviews addressing four overlapping areas. Because direct evidence for generative LLMs in adolescent suicide prevention remains limited, we also considered adjacent evidence from natural language processing and encoder-based classifiers. These studies were interpreted cautiously. They support the feasibility of detecting suicide-related language, but they do not establish that generative LLMs can safely conduct crisis intervention.

**Figure 2:**
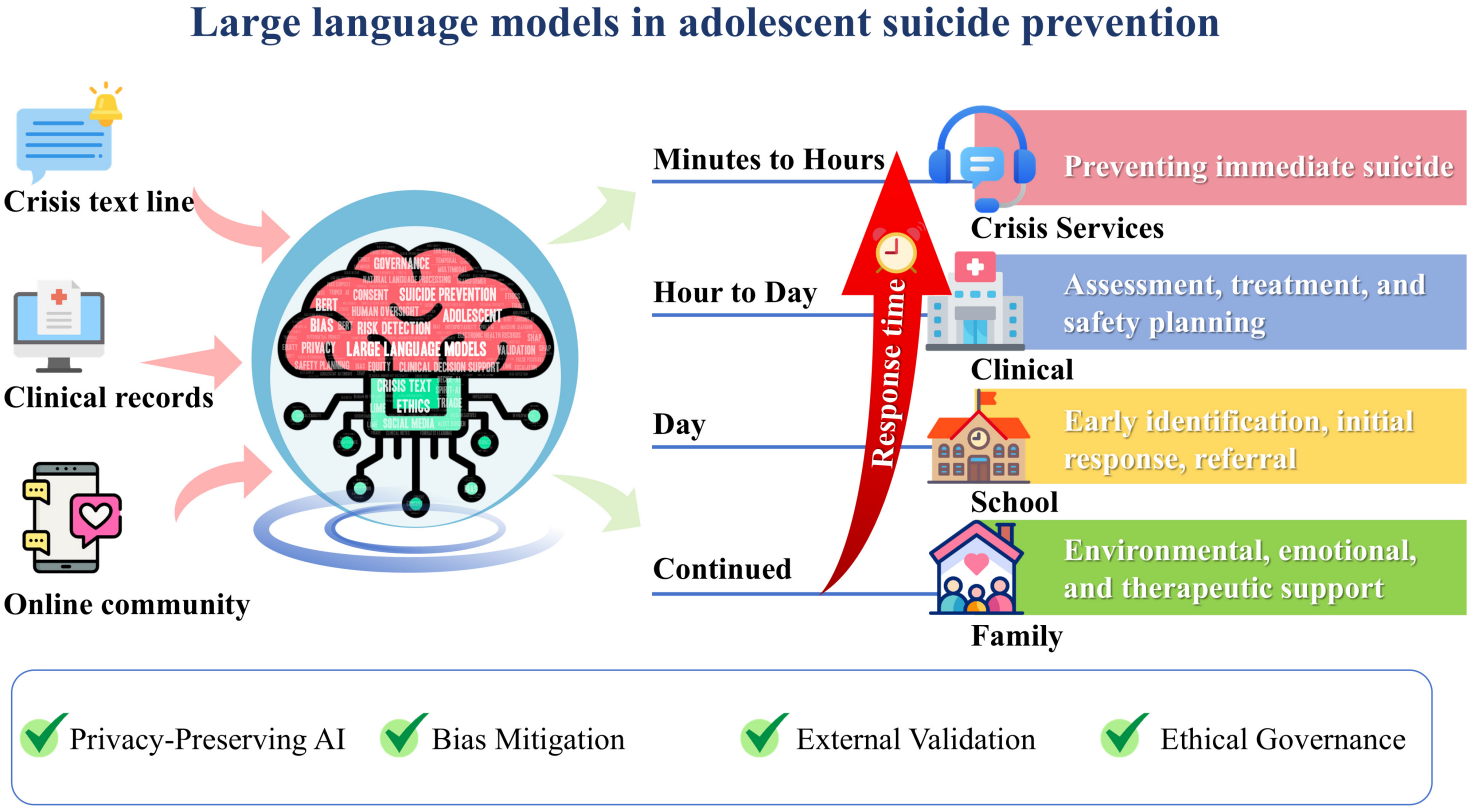
Evidence-to-Action Analytical Framework. The framework connects language signals to accountable action through three linked layers. Layer 1 identifies signal sources where adolescent suicide-related language originates: crisis chats, clinical records, school counseling, online disclosures, and family communications. Layer 2 classifies language tasks by evidence grade, which governs implementation readiness. Risk detection and flagging with encoder-based models (16, 17) carries established evidence (solid fill). Context summarization and extraction with mixed systems (19–22) carries emerging evidence (partial fill). Generative crisis intervention with generative LLMs (26–28) carries insufficient evidence (open fill). Evidence grades are defined as: Established, at least two prospective validations with adolescent data and outcome linkage; Moderate, retrospective validation in adolescent samples; Emerging, adult or single-platform evidence extrapolated to adolescents; Insufficient, conceptual or feasibility-only. Layer 3 specifies the safeguards and accountable outcomes required before deployment: technical calibration, governance protocols, and clinical oversight must lead to timely human review, proportional escalation, safety planning, linkage to care, and equity monitoring. The logical flow from signal detection through evidence grading to safeguarded action proceeds from left to right. Detailed mapping of representative studies to evidence grades and validation types is provided in Supplementary Table 2. Created with Microsoft PowerPoint and Weiciyun. Icons by Freepik from Flaticon, licensed under Flaticon License.

To organize the evidence, we used an Evidence-to-Action perspective. We therefore assessed the literature along three linked dimensions: the type of language task being performed, the practical setting in which the output would be used, and the safeguards needed before use with adolescents. This approach separates technical performance from implementation readiness. It also allows the review to identify where LLMs may be useful now, where the evidence remains premature, and which outcomes should be tested in future studies (Figure 2).

## Current evidence and its boundaries

The most developed work still comes from supervised classification with encoder-based models, particularly in crisis chats and hotline conversations, where the language is time-stamped and produced during acute distress. An explainable encoder-based classifier in youth crisis-text users showed that features such as self-reference, negation, low self-worth, and absolutist expressions could help identify suicidal ideation or high-risk behavior (16). Related hotline work has combined machine learning with psychological constructs to support real-time suicide-risk prediction during crisis chats (17). This work does not, however, prove that such models are ready for real-time decision-making in adolescent services. Many studies remain retrospective, use broad or unstable labels, and combine ideation, intent, and behavior in ways that may obscure clinically meaningful differences (18). For adolescents, this problem is especially relevant because suicidal communication can be indirect, developmentally shaped, and sensitive to the immediate social context.

Clinical text offers a second route for language-based support. Natural language processing models using unstructured clinical notes can improve suicide risk prediction beyond structured variables alone, and weakly supervised approaches have been used to identify current suicidal ideation in clinical documentation (19–21). More recent LLM-based systems have also extracted suicide-related social determinants of health, including housing instability, family conflict, trauma, substance use, and access barriers, from unstructured records (22). This type of extraction may help clinicians organize contextual information, but it should not be confused with validated prevention. Extracted risk or social determinants must still be linked to outcomes that matter. Without such linkage, information extraction remains closer to documentation support than to a demonstrated suicide-prevention intervention.

Generative LLMs raise a different set of questions. Their ability to summarize clinical context, translate technical language into developmentally appropriate explanations, or support protocol-based resource navigation may be useful in supervised workflows. In text-based counseling services, AI-assisted language analysis may support counselor feedback, service monitoring, and quality improvement (23–25). In suicide-specific settings, its role should remain supervisory rather than autonomous. These are plausible near-term uses because a human remains responsible for interpretation and action. Autonomous generative crisis intervention is not yet defensible for adolescent suicide prevention. Recent evaluations show that generative AI responses to suicide-related prompts contain method-related information, false reassurance, and hallucinated services (26), that LLM competency in evaluating appropriate responses varies across platforms (27), and that alignment between LLM outputs and expert clinician expectations remains uneven (28). For adolescent suicide prevention, this means that fluency cannot be treated as clinical competence. For adolescents, these risks carry distinct severity profiles. Hallucinated services or method-related information pose acute threats given developmental impulsivity. False reassurance and emotional dependence may delay caregiver engagement. Current evaluations show uneven alignment with expert expectations, and incidence rates of unsafe outputs remain unquantified in this population.

Social media data should be interpreted even more carefully. Adolescents may disclose distress online before contacting clinicians, school counselors, or crisis services, and studies of Reddit, Twitter/X, and other platforms have mapped suicide-related posts to themes such as hopelessness, perceived burdensomeness, harmful exposure, and protective messaging (29–31). Such evidence may inform public health surveillance, moderation support, or culturally sensitive outreach planning. Online adolescent language is shaped by slang, irony, memes, coded phrases, peer-group norms, and rapidly changing platform cultures (32). LLM-based psychological assessment through social media analysis has advanced risk-detection metrics by integrating prompt engineering with retrieval-augmented generation (33). Yet this work remains primarily adult-centric and lacks the adolescent-specific safeguards emphasized here.

Taken together, the literature shows that language models can identify, summarize, and structure suicide-related signals, but it rarely shows that these outputs improve adolescent outcomes. Future studies should report calibration, subgroup performance, alert burden, time to human review, appropriateness of escalation, safety-planning completion, linkage to care, and unintended harms.

Applying the Evidence-to-Action framework to existing studies illustrates its discriminative value. Work on youth crisis-text classification (16) and hotline prediction (17) demonstrates established signal detection, yet leaves implementation safeguards and accountable outcomes unaddressed. LLM-based information extraction from clinical records (22) shows technical feasibility but lacks linkage to safety-planning completion or care engagement. These examples reveal that technical performance alone does not constitute implementation readiness.

## Implementation and governance barriers

The main obstacle to using LLMs in adolescent suicide prevention is not only model accuracy. It is whether a system can be deployed without damaging trust, widening inequity, or producing alerts that no one can act on (34). Adolescents' language data are highly sensitive. These data can reveal distress, family conflict, trauma, substance use, abuse, sexuality, or self-harm thoughts. Routine de-identification is therefore insufficient. Data use must be justified by a clear prevention purpose, limited to the minimum necessary information, and governed by explicit rules on who can access the data, how long they are stored, and when confidentiality may be overridden (35–37).

Consent is especially difficult in adolescents. A model may be trained or deployed in settings where the adolescent, parents, school, platform, and clinician have different expectations about privacy and safety. Guardian permission alone may not protect the adolescent's trust, while adolescent assent alone may be insufficient when imminent risk requires family or emergency involvement. For this reason, LLM systems should specify consent procedures before data collection or deployment. These procedures should be written in developmentally appropriate language rather than buried in general data-use statements. In crisis contexts, confidentiality override rules must be narrow, transparent, and linked to human review.

Generalizability is another major barrier. Adolescents may express distress through slang, irony, memes, indirect farewell messages, or coded phrases that shift rapidly across cultures and peer groups. A high AUC in a retrospective dataset does not guarantee safe triage in a different service, especially when suicide attempts and deaths are rare outcomes and labels for ideation, intent, and behavior are often inconsistent (6, 7, 38). External validation should therefore include calibration, subgroup performance, and stress testing across age bands, language groups, sex, gender identity, neurodevelopmental status, and service setting. Average performance is not enough if the model fails in groups already facing barriers to care.

Bias is not limited to training data. It can enter through outcome labels, access to services, clinician documentation patterns, and the thresholds used to trigger alerts.

These concerns are consistent with broader evidence that health algorithms can reproduce inequities through proxy outcomes and EHR documentation patterns (39), that population health management tools may embed racial bias (40), and that medical LLMs can propagate race-based medicine (41). Adolescent suicide prevention also requires domain-specific reporting of consent, escalation rules, subgroup harms, and post-event review (42–45). A recent framework for evaluating LLM readiness in psychotherapy emphasizes that safety, confidentiality, equity, and effectiveness must be verified before clinical deployment, with explicit attention to escalation for suicidality and systematic adverse-event monitoring (46).

The most neglected problem is the gap between alert and action. A risk score has little value if it does not lead to a timely and proportionate human response. False negatives may delay care, but false positives may also harm adolescents through unnecessary emergency referral, privacy loss, family conflict, coercive intervention, stigma, or school discipline. Deployment should therefore define who receives an alert, how quickly it must be reviewed, what information is needed before escalation, and which actions are appropriate at different levels of concern. Automated emergency dispatch or mandatory caregiver notification should not be triggered by model output alone. To date, no published prospective deployment trial has measured alert-to-action latency, escalation appropriateness, or outcome linkage in adolescent LLM-supported systems. This section therefore identifies an evidence gap rather than a validated recommendation.

Accountability must also be assigned before deployment. LLM systems distribute responsibility across developers, platform operators, schools, clinicians, crisis services, and emergency responders. Without a defined chain of responsibility, each actor can assume that another actor owns the final decision. For adolescent suicide prevention, documentation should record model inputs, outputs, uncertainty, human review, and follow-up action. These records are not only legal safeguards. They are necessary for quality improvement and for detecting whether the system is causing delays, excessive escalation, or unequal harm.

Finally, LLM evaluation should be tied to prevention outcomes rather than only technical metrics. Safety planning and structured follow-up have evidence in suicide prevention, but an LLM is useful only if it strengthens the pathway toward such care (47–49). Prevention outcomes are closer to public mental health value than model fluency or retrospective classification performance. A system that detects more risk but overwhelms counselors, breaches trust, or delays response should not be considered successful.

## Research priorities for accountable deployment

Future work should move from retrospective model testing to prospective evaluation in adolescent-serving systems. The first priority is not simply to build larger models, but to create adolescent-specific evidence. New datasets should therefore include age-stratified adolescent language, culturally diverse expressions of distress, and outcome linkage to clinically meaningful events. Without such datasets, model performance will remain difficult to interpret and harder to translate into practice.

Model development should also become more clinically grounded. For many prevention tasks, smaller domain-adapted or more interpretable models may be preferable to very large proprietary systems (50–52). Geometric prompt optimization provides an efficient framework for structured prompting in engineering applications (53). Translating this optimization to clinical retrieval may strengthen protocol adherence in crisis settings. Even then, generative systems must be tested under adversarial and clinically realistic conditions, including coded language, method-seeking prompts, emotional dependency, and ambiguous intermediate-risk disclosures (26–28). The relevant question is not whether the model can sound empathic, but whether it behaves safely when the conversation becomes unstable.

The next research step should be implementation trials rather than more isolated benchmark studies. LLM-supported workflows could be tested in crisis text services, school mental health programs, pediatric emergency departments, or outpatient services, but only with human review preserved at safety-critical points. Trial outcomes should include time to human review, alert burden, proportional escalation, completion of safety planning, linkage to care, adolescent acceptability, and unintended harms.

Governance research must proceed alongside technical research. Current AI reporting standards can improve transparency, but adolescent suicide prevention requires additional details that are often missing (42–44). Post-deployment monitoring should track performance drift, subgroup inequity, unsafe generated content, delayed responses, and complaints from adolescents or families. These processes should not be treated as administrative extras. In a safety-critical setting, they are part of the intervention.

A realistic research agenda should therefore test a small number of clinically important hypotheses: whether LLM summaries improve human assessment compared with risk scores alone; whether protocol-grounded generation reduces unsafe responses; whether subgroup calibration reduces inequitable escalation; and whether LLM-assisted triage improves linkage to care without increasing avoidable harms. These questions connect model development to public mental health value. They also keep the field focused on accountable deployment rather than technological novelty.

## Discussion

LLMs offer a plausible but bounded opportunity in adolescent suicide prevention. Their most defensible role is not to diagnose, counsel, or manage suicidal crises independently, but to help human services notice and organize risk-relevant language that might otherwise remain fragmented across multiple sources. This distinction is important. In suicide prevention, a fluent answer is not the same as a safe intervention, and a high-performing classifier is not the same as an accountable care pathway.

The central question is therefore whether language models can improve human response. This requires a shift in evaluation. Retrospective discrimination metrics are necessary but insufficient. Prevention outcomes are closer to public mental health value than model fluency or benchmark performance.

This mini review uses this practical boundary to interpret the current evidence. Current evidence is strongest for supervised risk-signal flagging and more preliminary for contextual summarization, extraction of suicide-related information, and counselor-support functions. It does not yet support autonomous generative crisis intervention for adolescents.

Several claims herein are extrapolated from adult electronic health records, English-language crisis chats, single-platform social media, or encoder-based classifiers. These are labeled as adjacent evidence supporting conceptual feasibility and should not be interpreted as direct proof of clinical readiness for adolescent generative LLM deployment.

Several limits should be acknowledged. This is a targeted mini review rather than a systematic review, and the evidence base is moving quickly. We included studies on natural language processing and encoder-based classifiers because direct evidence for generative LLMs in adolescent suicide prevention remains limited. The proposed Evidence-to-Action perspective is also intended to guide evaluation, not to justify immediate implementation. A further limitation concerns the Evidence-to-Action framework itself. This framework is a conceptual analytical model developed by the authors to organize existing evidence and identify implementation gaps. It has not been subjected to expert consensus validation, stakeholder consultation, or empirical testing against established implementation frameworks such as RE-AIM or the readiness criteria proposed by Stade et al. (46) for LLMs in psychotherapy. Its purpose is to structure current evidence and guide future research directions, not to prescribe validated implementation standards. It should be regarded as an evaluative heuristic framework rather than an established theoretical model.

For now, LLMs should remain safety-critical adjuncts to human care. Any move toward LLM-initiated outreach, real-time safety coaching without concurrent human review, or automated emergency escalation would require prospective validation, explicit pediatric oversight, transparent liability, and developmentally appropriate consent. Until then, the responsible path is narrower but more defensible: use LLMs to support accountable human action, not to replace it. Consistent with the mini review format, these conclusions are scoped to guide future research and policy, not to establish clinical implementation standards.
