# Supplementary material for: Large language models in adolescent suicide prevention: from language signals to accountable action
Source: Front Public Health. 2026 Aug 3;14:1872450. doi: 10.3389/fpubh.2026.1872450 (PMC13477962; doi:10.3389/fpubh.2026.1872450)
Supplement: Supplementary file 1 [file Table_1.docx]

**Supplementary Table 1** **| Comparison with previous reviews on artificial intelligence and large language models in suicide prevention.**

| Dimension | Levkovich et al. [13] | Li et al. [14] | Holmes et al. [15] | Present review |
| --- | --- | --- | --- | --- |
| Primary scope | Technical comparison of BERT-based and LLM classifiers | AI applications and challenges in adolescent suicide prevention | Scoping review of LLM applications in general suicide prevention | Evidence-to-Action translation for adolescent suicide prevention |
| Population focus | General population | Adolescents as one subgroup among broader youth | General population | Adolescents aged 10-19 years as the primary focus |
| Evidence grading | None | None | None | Four-tier grading from Established to Insufficient |
| Implementation and governance linkage | Not systematically addressed | Briefly mentioned | Not systematically addressed | Core focus: safeguards, accountability, escalation, and equity |

This comparison is limited to the three most directly relevant previous reviews cited in the manuscript. Other syntheses address broader digital mental health or machine learning topics outside the scope of this mini review.
