# Supplementary material for: Large language models in adolescent suicide prevention: from language signals to accountable action
Source: Front Public Health. 2026 Aug 3;14:1872450. doi: 10.3389/fpubh.2026.1872450 (PMC13477962; doi:10.3389/fpubh.2026.1872450)
Supplement: Supplementary file 2 [file Table_2.docx]

**Supplementary Table 2** **| Mapping of representative studies to evidence grades and validation types.**

| Representative study | Data source | Study design | Population | Validation type | Evidence grade | Grading rationale |
| --- | --- | --- | --- | --- | --- | --- |
| Thomas et al. [16] | Youth crisis text | Development and validation | Adolescents | External validation with outcome linkage | Established | Adolescent sample; validated classifier; linked to high-risk behavior outcomes |
| Grimland et al. [17] | Crisis hotline chats | Real-time prediction model | Adolescents | Real-time validation with psychological construct integration | Established | Adolescent sample; real-time risk prediction during crisis chats; integrated psychological factors |
| Levis et al. [18,19]; Cusick et al. [20] | Clinical notes | Retrospective EHR analysis | Adult / mixed | Retrospective validation | Emerging | Adult EHR data; supports technical feasibility; extrapolation to adolescents requires caution |
| Wang et al. [21] | Clinical records | Information extraction framework | Mixed | Extraction without prevention outcome linkage | Emerging | Demonstrates LLM-based extraction of social determinants; no validated linkage to safety planning or care engagement |
| Campbell et al. [32] | Generative AI responses | Content analysis | Not adolescent-specific | Safety evaluation | Insufficient | Identifies method-related information and false reassurance in generated responses; no adolescent data or prospective deployment testing |
| McBain et al. [33,34] | Multiple LLM platforms | Comparative alignment study | Not adolescent-specific | Expert-clinician expectation comparison | Insufficient | Evaluates competency and alignment across platforms; uneven agreement with expert expectations; unsafe output incidence unquantified in adolescents |

Evidence grades follow the definitions provided in the main text. This table maps the studies explicitly cited in Figure 2. Studies [18,19] share a common first author and design; [33,34] share authors and platform. The grade for [17] reflects its grouping with [16] as established signal detection in Figure 2. The grade for [32-34] reflects the manuscript assessment that generative crisis intervention evidence is much weaker than encoder-based detection.
